# Isolating reactive metal-based species in Metal–Organic Frameworks – viable strategies and opportunities

**DOI:** 10.1039/d0sc00485e

**Published:** 2020-03-25

**Authors:** Rosemary J. Young, Michael T. Huxley, Emilio Pardo, Neil R. Champness, Christopher J. Sumby, Christian J. Doonan

**Affiliations:** Department of Chemistry, Centre for Advanced Nanomaterials, The University of Adelaide Adelaide Australia; School of Chemistry, The University of Nottingham Nottingham UK christopher.sumby@adelaide.edu.au christian.doonan@adelaide.edu.au; Institute of Molecular Science, University of Valencia Valencia Spain

## Abstract

Structural insight into reactive species can be achieved *via* strategies such as matrix isolation in frozen glasses, whereby species are kinetically trapped, or by confinement within the cavities of host molecules. More recently, Metal–Organic Frameworks (MOFs) have been used as molecular scaffolds to isolate reactive metal-based species within their ordered pore networks. These studies have uncovered new reactivity, allowed observation of novel metal-based complexes and clusters, and elucidated the nature of metal-centred reactions responsible for catalysis. This perspective considers strategies by which metal species can be introduced into MOFs and highlights some of the advantages and limitations of each approach. Furthermore, the growing body of work whereby reactive species can be isolated and structurally characterised within a MOF matrix will be reviewed, including discussion of salient examples and the provision of useful guidelines for the design of new systems. Novel approaches that facilitate detailed structural analysis of reactive chemical moieties are of considerable interest as the knowledge garnered underpins our understanding of reactivity and thus guides the synthesis of materials with unprecedented functionality.

## Abstract 2

Metal–organic frameworks can be used to stabilise reactive metal-based species, uncover new reactivity, and investigate metal-centred reactions responsible for catalysis.

## Graphical abstract

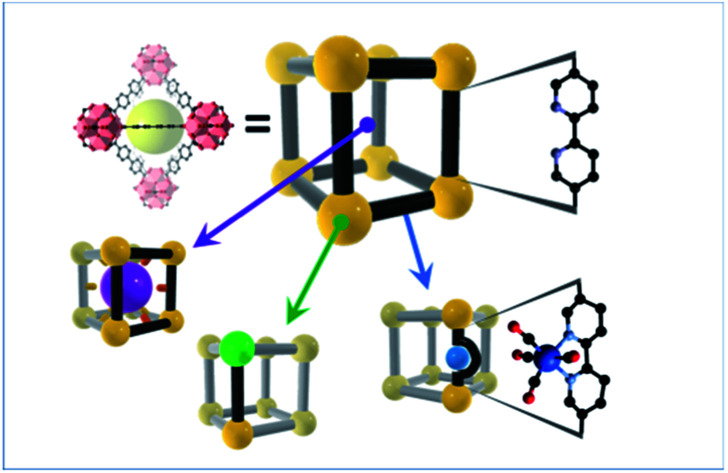

## 1. Introduction

The structural elucidation of reactive molecular species provides valuable insight into bond activation mechanisms that underpin areas of fundamental scientific and commercial interest such as catalysis and chemical synthesis.^1–4^ For example, the active forms of many homogeneous organometallic catalysts are coordinatively unsaturated species that are realised *via* ligand dissociation processes. Characterising such reactive intermediates *in situ* is challenging due to their short solution lifetimes^5,6^ and typically requires kinetic trapping *via* low temperature matrix isolation strategies,^7,8^ and/or studied by laser-pulsed time-resolved spectroscopic techniques that allow rapid interrogation of the chemistry.^9–11^ One approach to enhance the stability of a reactive species, to facilitate structural characterisation, is *via* encapsulation within the cavity of a host.^12–14^ Examples of molecular capsules as hosts to trap reactive entities are well-established, dating back to Cram and co-workers who, for example, demonstrated the isolation of cyclobutadiene within a carcerand.^15^ In subsequent years the constrained microenvironments within molecular cages^16^ have been shown especially suitable for stabilising reactive organic,^17^ inorganic^18^ and organometallic^19,20^ species. Indeed, this research has endeavoured to understand, and control, the supramolecular interactions that underpin the specific kinetic and thermodynamic factors that engender the stabilisation of guests. Such studies have led to the synthesis of artificial catalysts that replicate the efficiency and selectivity of enzymes.^17,21–23^

Metal–Organic Frameworks (MOFs) are a class of extended materials composed of metal-based nodes (ions, or clusters) connected *via* organic links.^24–26^ MOFs typically show high permanent porosity and, as a result, have been extensively investigated for their gas adsorption and separation properties.^27–29^ However, their open pore networks also offer excellent opportunities as solid-state matrices for the isolation, stabilisation and structural characterisation of reactive species. This is due to the molecular level control of their structural chemistry that is facilitated by a building-block synthetic approach. For example, the size, shape and chemical functionality of their pore cavities can be tailored to anchor inorganic or organometallic compounds to their framework,^30,31^ isolate guests *via* supramolecular forces or confine molecules through steric effects.^32^ The periodic nature of the host framework isolates reactive metal sites, thereby negating the need to use sterically demanding ligands to prevent ligand disproportionation, decomposition and nanoparticle formation; thus, similar to matrix isolation methods the reactive entity is predominantly stabilised through kinetic factors. These design features are, generally, coupled with a high degree of crystallinity that allows for X-ray diffraction methods to be employed as a technique for the structural elucidation of guests.^33–36^

**Fig. 1:**
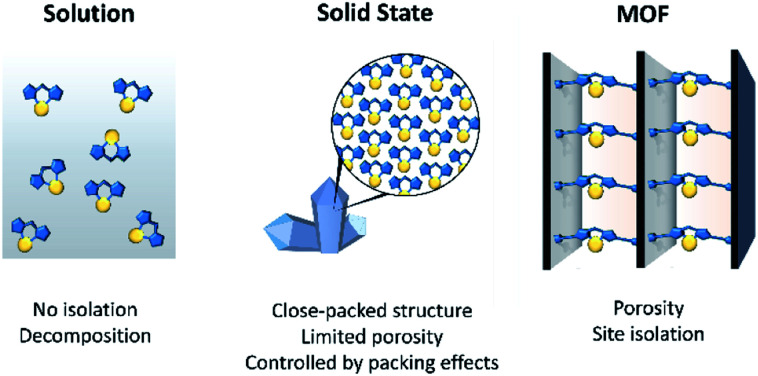
A schematic showing a comparison of the chemical environment of reactive metal complexes in solution, in the solid-state as a typical molecular crystal, and isolated in a MOF support.

The concept of using MOFs as crystalline scaffolds for the characterisation of guests is established. There are several examples where single crystal X-ray diffraction (SCXRD) has been employed to structurally identify the initial stages of gas adsorption,^37^ MOF-adsorbate binding sites^38^ and molecules absorbed from solution.^34,36,39,40^ However, there is significant scope for the development of MOFs as isolation matrices for metal-based moieties that are unstable in solution (Fig. 1). Indeed, the potential of MOFs to facilitate such chemistry is illustrated by analogous studies that have explored excited state chemistry and reactions of discrete organometallic compounds in the crystalline state.^41–45^ Research in this related area of molecular solid-state materials has shown that highly reactive species can be stabilised in the absence of solvent, indicating significant opportunities for the study of such solid-state “matrix-isolated” reactive species.^46^ A salient example, from a field which will be explored in more detail below, was reported by Weller and co-workers who elucidated a single-crystal to single-crystal (SC–SC) process that led to the X-ray structure determination of an elusive transition-metal σ–alkane complex.^47^ However, a limitation of molecular crystals is that porosity, which is required for diffusion of reactants to active sites, has yet to be reliably controlled or predicted. This has obvious implications for the general application of this approach and for the exploration of such systems as catalysts. In contrast to molecular crystals, the permanent porosity of MOFs, and hence improved guest diffusion, allows for the solid-state environment to be fully capitalised on.

This perspective article canvasses the extensive scope for studying chemistry in MOF pores with a focus on how the crystalline architectures convey significant structural insight to metal-based and organometallic species isolated within their frameworks. Prior art, with respect to similar entities trapped and studied within solid-state lattices, is outlined to establish context, then the properties of molecular crystals are contrasted with those of MOFs. Strategies by which inorganic and organometallic species are docked within MOF materials are a necessary part of the process, and are thus identified. These include: substitution into the metal node, tethering to either the metal node, or the organic linker, and isolation *via* supramolecular forces or confinement of molecules through steric effects in MOF pores. Having considered synthetic approaches, we further discuss salient examples that demonstrate how MOFs can help afford significant structural insight into the structures and chemistry of inorganic and organometallic species, can be used to characterise reactive intermediates, and provide insight into reactivity. In some instances, X-ray crystallography fails to impart structural insight and we also discuss these examples to highlight the limitations of the approach. It is worth noting that these topics intersect with aspects of MOF chemistry concerning the formation of coordinatively unsaturated nodes for reversible small molecule binding (*e.g.* gas adsorption), and the structural characterisation of small organic molecules bound within their pore network. Given the breadth of these topics and significant attention they have received, we have specifically excluded this work, and direct the reader to relevant contributions on small molecule characterisation at coordinatively unsaturated nodes in MOFs^38,48–50^ and work regarding structural characterisation of small molecules in frameworks.^34,36^ Finally, we would like to clarify the term ‘reactive species’; reactive, like the term 'stable', is widely used but often not well defined. Broadly speaking, chemicals that react vigorously or release significant energy upon reaction generally meet the definition of reactive. In this perspective we have used this term more generally to encompass species that are difficult or challenging to isolate in the solid-state as discrete entities, *e.g.* those that might dimerise if not retained in a framework, which might readily undergo ligand exchange upon crystallisation or are insufficiently long-lived to allow characterisation *via* routine spectroscopic and diffraction methods.

## 2. Matrix isolation of metal-based species in the solid-state

Coordination complexes and organometallic compounds have traditionally been studied in solution wherein their behaviour and activity has been extensively investigated and developed over the past century.^51–53^ Reactions of well-defined metal complexes and “matrix isolation” of reactive species in the solid-state have received more limited attention, mostly due to the great difficulties involved in both performing the reactions and characterising the reaction process.^46^ However, if these challenges are overcome, solid-state chemistry presents unique opportunities to study highly reactive species and garner insight into reaction mechanisms, thereby improving selectivity, achieving higher yields and gaining access to reactions which are not feasible to study in solution.^46,54–57^ These insights also extend to chemistry in solution or the gas phase (heterogeneous catalysis). A number of reviews have been published on this topic,^45^ including a recent comprehensive account by Pike and Weller.^46^ Thus, we focus only on a few examples to highlight the specific challenges involved in studying reactivity in the solid-state, allowing comparison of the opportunities that permanently porous MOFs afford as a counterpoint to molecular solid-state species. At this point it is worth differentiating matrix isolation in the solid-state from the more traditional process of isolating species in a frozen glass. Traditional matrix isolation experiments allow kinetic trapping of unstable species at low temperatures whereas isolation in solids relies on both kinetic trapping of the species in the solid and thermodynamic contributions from the surrounding crystal or framework material. Moreover, the extent of both these contributions varies from system to system.

The challenges involving reactions of metal complexes in the solid-state can be expressed simply in terms of three factors; characterisation, stability and diffusion. Firstly, in order to study reactions of metal complexes in the solid-state, understanding the limitations of characterisation techniques is essential. Single crystal X-ray diffraction (SCXRD) is the pre-eminent technique for elucidating the structure of a solid-state material; however, this approach requires retention of crystallinity, high levels of conversion, and stability of the complexes for the lifetime of the diffraction experiment. The latter point is a lesser objection given the rapid collection times afforded by modern diffraction equipment and synchrotron facilities. For single crystal-to-single crystal (SCSC) transformations to occur, long-range order must be retained following the reorganisation of atoms that arises during the reaction. Typically for molecular crystals, only small structural reorganisations can be tolerated without loss of crystallinity, unless the sites around which the reaction occurs do not affect the integrity of the crystalline lattice. Weller^46^ suggests that, in general, there is a limit of *ca.* 15% of volume change which can be tolerated for transformation in a molecular organometallic crystal, which is supported by other studies.^58^ Although spectroscopic analysis, including solid-state NMR spectroscopy, is feasible in many instances, problems can arise due to incomplete reactions, as a result of poor reactant diffusion, and formation of a multitude of different products from complex reaction pathways.

The stability of reactive metal species is typically aided by the confined environment of the solid-state structure, which prevents attack by coordinating species (including solvent) or loss of weakly bound ligands. A particular advantage of having the reactive metal species anchored within a solid-state lattice is that the formation of dimers, or clusters between the complexes is inhibited. However, their relative proximity can also be disadvantageous as diffusion of reactants throughout the lattice can be impeded. Furthermore, different reaction kinetics at the crystal surface compared to the interior can obfuscate a detailed analysis of structure and reaction rates. Finally, the surface of crystals can become passivated by a layer of product, slowing the progress of the reaction and inhibiting diffusion of reagents to the interior of the crystal.^46,56^

**Fig. 2:**
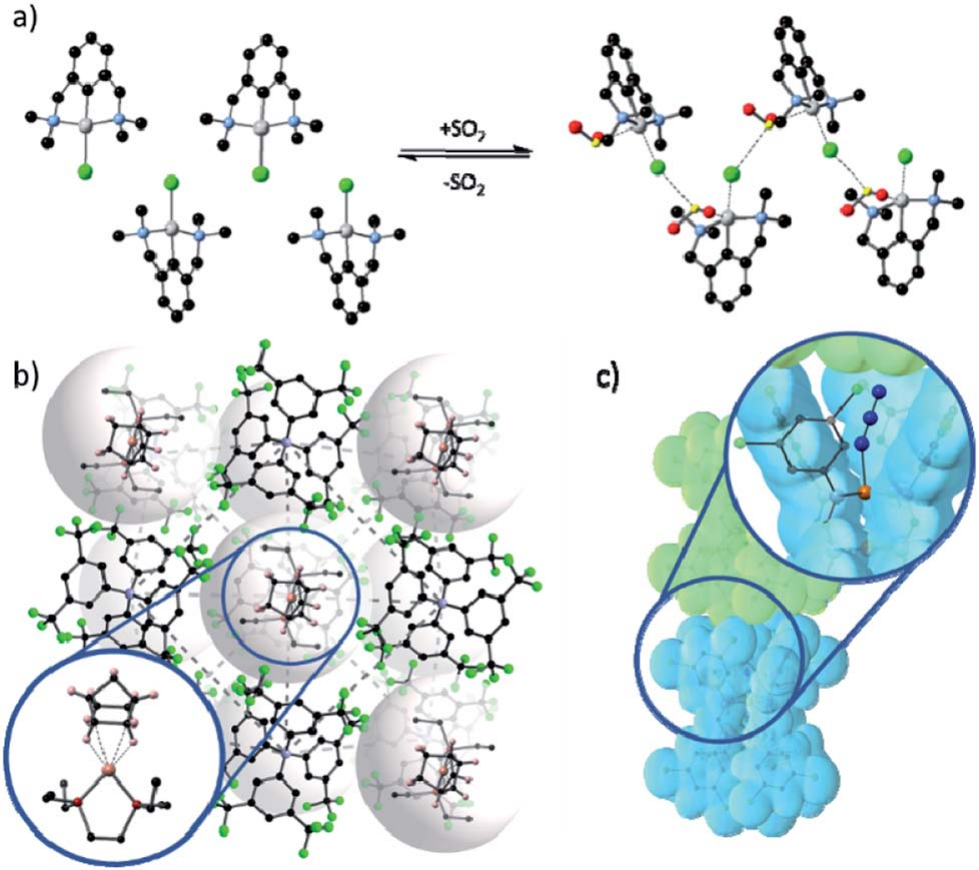
Examples of metal based species have been isolated in the solid-state through the use of (a) conformationally constraining ligands, as demonstrated by van Koten's platinum pincer complexes coordinating to SO_2_ and stabilised by a β-type bonding network (Pt – silver; C – black; N – blue; Cl – green; O – red; S – yellow);^58^ (b) bulky anions forming a stable lattice with cavities in which the cation is confined and prevent the departure of the coordinating alkane (Rh – pink; P – dark red; C – black; B – blue; F – green);^47^ and (c) sterically bulky ligands form a reaction pocket in which azide photo-cleavage can occur without disrupting the crystal lattice (Ru – orange; N – blue; C – grey; the van der Waals surface of adjacent complexes is shown in blue and green).^59^

Despite these challenges, reactions of metal complexes in the solid-state have been successfully achieved for a select, but highly interesting, series of studies. For example, van Koten^58,60^ demonstrated controllable and reversible addition of sulfur dioxide gas to platinum complexes arranged in a crystalline lattice, which caused a *ca.* 15% expansion in the unit cell volume. Tridentate pincer ligands forced the coordination of the gas in an unusual conformation, and the lattice was stabilised by the presence of a β-type bonding network (Fig. 2(a)).^58^ Monitoring of the conversion showed inwards travel of the reaction from the surface of the crystal, and although the crystal shape was retained, the single crystal converted into a multi-crystal composite.

Using bulky ligands or anions, which dominate the packing, can create a rigid pseudo-framework within which small modifications around the metal centre can be tolerated. This approach^47^ was used to demonstrate, for the first time, the formation and isolation of a sigma-bonded alkane rhodium complex in the solid-state. Furthermore, this species was shown to be stable at room temperature with a lifetime of several minutes. These results were achieved by using a tetrakis[3,5-bis(trifluoromethyl)phenyl]borate ([BAr^F^_4_]^−^) anion, which packs to form a stable lattice. The packing of these bulky anions provides well-defined pockets in which the sigma-bound complex is stabilised and hence is observable by SCXRD (Fig. 2(b)). This system has been used by Weller to explore the effects of modifications to the cation ligand^61–63^ and changing rhodium for iridium,^64^ as well as demonstrating selective C–H activation^65^ and light hydrocarbon catalysis.^66^ Weller's approach allows highly unstable complexes to be sterically trapped within a crystalline lattice, facilitating the characterisation of the resulting species. The overall robustness of the crystalline lattice is engendered by the rigid packing of the bulky anions, allowing for changes in the coordination environment of the metal centre without loss of crystallinity. An alternate approach described by McKeown *et al.*^67^ showed that solvent accessible channels can also facilitate reactions in the solid-state by using a non-coordinating solvent to introduce bidentate ligands into a crystal lattice, where they coordinate adjacent metal centres, forming a coordination polymer and stabilising the lattice upon removal of solvent. Supramolecular organisation of molecular hosts has also been used to provide space for *E*-to-*Z* photoisomerisation reactions about Zn(TA)_2_(H_2_O)_2_ complexes (HTA = tiglic acid);^68^ the cavity of the *C*-ethylcalix[4]resorcinarene molecule provides void space to accommodate the TA rearrangement.

Additionally, Brookhart^56^ has demonstrated the selective hydrogenation of ethylene relative to propylene by iridium catalysts in the solid-state. The iridium cations were complexed by a bulky, electron-deficient, pincer ligand that forms a reaction pocket in which the reaction could occur. Careful ligand choice was essential, with the use of less hindered ligands resulting in the formation of dimers. In the solid-state, the complexes pack to form channels running along the *a* and *b* axes, which are filled with disordered solvent molecules. These solvent-filled channels are instrumental in allowing the diffusion of gaseous ligands to the metal centres. The reaction pocket also prevents the formation of longer chain alkenes due to steric constraints, and as this is effective only in the interior of the crystal, the surface was pacified intentionally with CO to block surface reactivity and promote selectivity.

Other reactions, which require little change in overall molecular shape such as those based upon photo-initiated rearrangements,^44,69,70^ or ligand cleavage,^59^ can also be observed in the solid-state. Powers' study^59^ of a Ru–azide complex elegantly demonstrates the latter approach. Photoexcitation leads to cleavage of the azide ligand, liberating N_2_ and leading to the formation of a Ru–nitride complex. The system design provides a coordination pocket which hosts the azide ligand such that when photocleavage occurs there is minimal change in the geometry or conformation of the complex ensuring a SCSC transformation occurs (Fig. 2).

These studies demonstrate that to isolate interesting reactive species and/or facilitate their solid-state reactions, it is important to design crystalline systems with a number of key features. First and foremost, accessible pore networks are imperative to enable access to the reactive sites. This is most readily achieved through a porous network; however, gas diffusion experiments have shown that small molecules can diffuse through ostensibly non-porous materials. Nevertheless, recent studies on a single molecule catalyst by Weller clearly showed the importance of porosity to reaction kinetics,^61^ while other work by Brookhart, among others, has highlighted that constrained environments may allow for selectivity not realised in solution.^56,59,65^ These observations clearly indicate that a balance needs to be achieved between providing open networks to access the reaction centre and a constrained environment that stabilises species and directs reactivity. Secondly, reactions in the solid-state should occur with limited molecular movement, particularly avoiding changes that lead to large modifications of crystal packing. Thirdly, (near) complete conversion must occur to facilitate characterisation as the solid-state does not allow for isolation and purification processes. Moreover, complete conversion, combined with minimised molecular motion during and after the reaction sequence, can help to maintain crystallinity, thereby allowing investigation by SCXRD. The open pore networks of MOFs readily meet many of these criteria. Molecular level control of their structural chemistry is imbued by a building-block synthetic approach,^71^ which allows both the tailoring of the pore network to anchor inorganic or organometallic compounds, and controllable access to these reactive sites without structure disruption. Certain MOFs have inherent flexibility^72,73^ and can tolerate significant structural changes. Furthermore, these design features are, generally, coupled with a high degree of crystallinity that allows for SCXRD to be used to characterise intermediates and products.^33,39^ Compared to molecular crystals, MOFs also provide a high degree of chemical and thermal stability^74^ allowing reactions, which might not be tolerated in a crystalline lattice stabilised by intermolecular forces, to occur without host decomposition.

## 3. General approaches to matrix isolation in MOFs

The ability to structurally isolate and stabilise reactive complexes (matrix isolation) within a MOF's pores commonly requires post-synthetic metalation (PSMet)^30,31^ or more general post-synthetic modification (PSM)^75,76^ strategies. These processes allow manipulation of the MOF structure post-synthesis to unveil metal ion binding functionality, and to facilitate the incorporation or exchange of metal ions. MOFs are commonly prepared under solvothermal procedures, which involve conditions of elevated temperatures, coordinating solvents and the addition of small quantities of acids or bases, chemistry that is typically incompatible with reactive metal complexes or nanoclusters. Even alternative synthetic conditions such as room temperature syntheses, ball milling and flow chemistry procedures are not readily compatible with reactive metal entities due to exposure to coordinating solvents, moisture and oxygen. Thus, a variety of PSMet strategies need to be employed to install or generate a reactive metal site, although, in certain instances, stable moieties can be introduced during MOF synthesis (either as part of the metal node or appended to an organic link) and then their reactivity revealed by PSM.

**Scheme 1:**
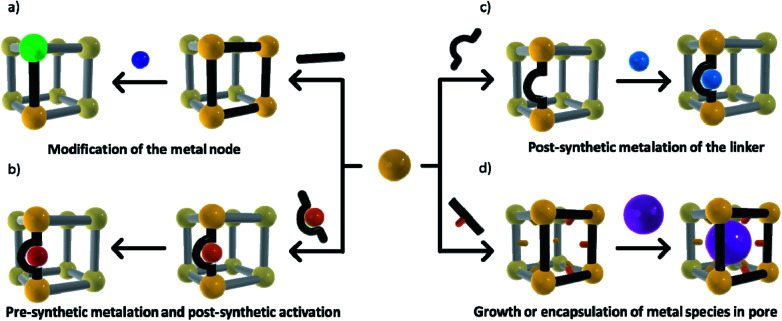
Matrix isolation methods in MOFs can be achieved *via*; (a) node related PSM to facilitate attachment of a species, or complete cation metathesis/PSM; (b) PSMet at preinstalled linkers or those unveiled in the MOF post-synthetically; (c) installation of a pre-metalated linker and subsequent chemistry to reveal reactivity; and (d) utilising the pore chemistry to stabilise molecular complexes or subnanometre metal cluster.

The chemical diversity of the MOF components (nodes and linkers) is critical for facilitating matrix isolation. The metal nodes, particularly those coordinated by non-structural ligands, can be used as sites for incorporating or anchoring metal species to the MOF. Additionally, metal binding groups can be an intrinsic part of the organic linkers or they can be post-synthetically introduced. Finally, the MOF pore environment can be designed to bind and stabilise metal-based guests *via* supramolecular forces or confinement effects. A defining feature of the strategies outlined below is that the chemistry occurs with the retention of crystallinity, thus facilitating structural insight. These approaches are grouped based on whether they occur at the node, linker or within the pore network (Scheme 1) and include:

(a) Modification of the node, such as cation metathesis at the metal node to give a reactive species, or PSMet of the node to append a metal complex;

(b) A pre-metalation approach and subsequent chemistry to reveal the reactive metal centre;

(c) PSMet at preinstalled linkers or those unveiled in the MOF post-synthetically; and,

(d) Growth or encapsulation of metal nanoparticles, clusters or metal complexes within the pores of the framework.

Alongside considerations of the strategy by which reactive metal centres are introduced, thought needs to be given to the methods of characterisation. Despite numerous examples of PSM and PSMet in MOFs, structural characterisation of the incorporated metal complexes and, in particular, their subsequent chemistry, remains rare. The importance of retaining crystallinity is that X-ray crystallographic studies can be applied to determine the structure of the metal species. As noted, this technique is considered the ‘gold-standard’ of structural determination as it provides precise atomic-scale information that is crucial to understanding chemical reactivity. The diverse chemistry encompassed in Scheme 1, coupled with the unique properties of a given MOF material, preclude a general strategy for ensuring crystallinity throughout the PSM processes. Nevertheless, from consideration of the literature in subsequent sections, we have identified some guiding principles that facilitate the structural characterisation of reactive species isolated in MOF pores.

(i) *Using frameworks that possess low symmetry space group to reduce the chance that the metal guest is located on a special position and its structure disordered over this site*. As outlined in Section 4a, this is a considerable challenge for strategies where the metal node is the site of the reactive metal centre as many MOFs crystallise in high symmetry space groups with the metal nodes on these special positions.

(ii) *Having frameworks comprising structurally flexible organic linkers allows for varied bite angles and geometries at the extraneous metal centre without introducing strain on the framework and loss of long-range order*. This is especially useful for PSMet approaches outlined in Section 4b. An interesting counterpoint to this is that a rigid framework site could impart bond strain on the metal centre which distinguishes the chemistry from that encountered in solution. Indeed, unique coordination environments enforced by the rigid MOF architecture have been structurally elucidated at the metal nodes.^31^ Nevertheless, while framework flexibility appears to facilitate structural characterisation by SCXRD, inherently rigid MOF matrices can be employed to enhance reactivity, thereby benefitting catalysis.

(iii) *Avoiding steric crowding of the pores by ensuring the MOF structure does not exhibit too high a density of guest metal sites resulting in non-quantitative conversion*. This latter point can be mitigated by diluting the donor sites by substitution of a blank linker but this is to the detriment of structural characterisation by SCXRD.

Having elucidated some general principles, the following sections provide examples of where reactive metal-based species can be site isolated and stabilised within a MOF network, and furthermore, structurally characterised to provide novel chemical insight into unstable or unusual structures or metal-centred reactivity. The examples exemplify the guiding principles outlined above and are chosen to provide insight into how to use MOFs to isolate reactive species and inform the design of new catalysts or solid-state reactions.

## 4. Exemplars of matrix isolation in MOFs

### (a) MOF node matrix isolation

The metal nodes of MOFs have served as sites for forming and isolating unusual metal clusters through selective and/or partial metal cation substitution,^77,78^ or for studying chemically unique metal centres within as-synthesised nodes.^79^ Furthermore, the MOF nodes can also be an anchoring point for appending metal centres to an existing node.^80^ The chemistry of selective and/or partial metal cation exchange at MOF nodes has been canvassed in an excellent review by Brozek and Dincă in 2014.^31^ In this work the authors noted that, through selective metal exchange at the nodes, structures impossible to access in solution could be achieved. However, one of the significant challenges of this approach is that structural insight is difficult as the metal nodes are often located on high symmetry sites within the unit cell of the structures. This averages out the coordination environment of the post-synthetically added metal centre with the atoms of the original node. Mostly, in these cases, structural insight comes from allied spectroscopic and X-ray scattering experiments and not from single crystal diffraction experiments. Despite this, we include examples of this chemistry because it points to opportunities that can be grasped if lower symmetry MOFs are employed; we further expand this concept in Section 5 where we provide a number of guidelines to enable the more widespread utilisation of this approach.

**Fig. 3:**
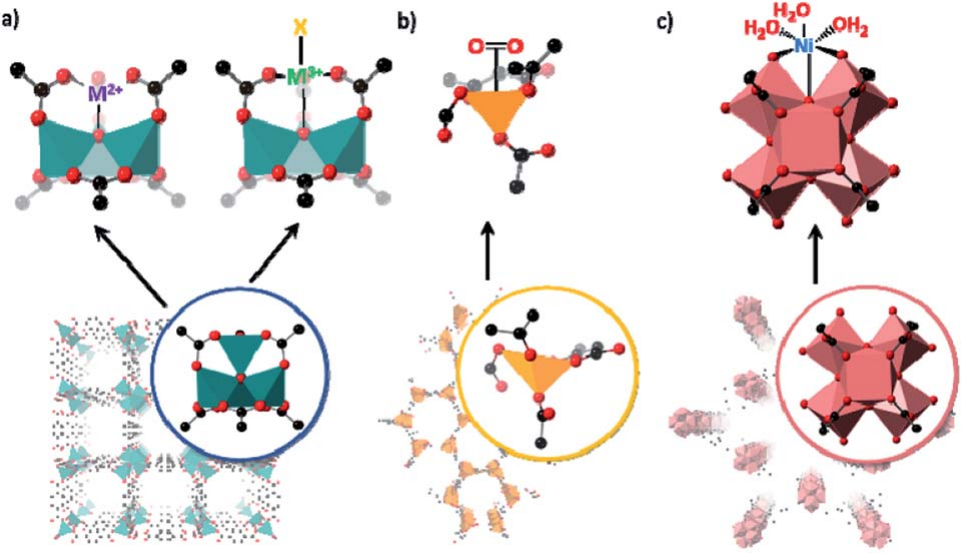
Simplified representations of matrix isolation at the MOF nodes including: (a) selective cation exchange of the Zn_4_O nodes of MOF-5 with a range of transition metals in the +2 and +3 oxidation states, allowing the formation of unusual coordination environments (Zn – teal; O – red; C – black); (b) the formation of coordinatively unsaturated nodes in MOF-74 allowing oxygen binding at low temperatures (Fe – yellow; O – red; C – black); (c) and the introduction of nickel atoms at the zirconium nodes of NU-1000 by atomic layer deposition (Zr – pink; O – red; C – black).

A seminal example of how cation exchange can be used to trap metal ions in unusual coordination geometries at a MOF node was reported by Brozek and Dincă.^81^ In this work the authors showed that Ni^2+^ could be exchanged for Zn^2+^ at the Zn_4_O nodes of MOF-5 (Zn_4_O(BDC)_3_ (BDC = benzenedicarboxylate)) (Fig. 3). The rigid MOF structure maintained a tetrahedral environment for the node cations thus enforcing an unusual pseudo-tetrahedral oxygen ligand arrangement around Ni^2+^. The Ni^2+^-exchanged MOF was characterised by spectroscopic methods but due to the high symmetry of the MOF, structural analysis using X-ray crystallography was precluded. Nonetheless, this result set the scene for further studies to isolate coordinatively unusual species. This strategy has been used to incorporate other first row transition metals into MOF-5 including Cr, Fe and Mn and explore unconventional chemistry.^38,41,44^ Although high symmetry precluded SCXRD analysis of cation exchanged metal nodes, an advantage of using MOF-5 is that the extraneous transition metal has spectroscopic and electronic signatures that are distinct from the spectroscopically silent Zn^2+^ cations.

Dincă *et al.* employed an analogous approach to install Ni^2+^ sites at the nodes of MFU-4l (Metal–Organic Framework Ulm University-4l).^82^ The partial substitution of the Ni^2+^ ions at the periphery of the node (1–30% Ni^2+^), afforded a MOF that showed excellent catalytic performance for the dimerization of ethylene to 1-butene.^83^ In other examples, Co^II^ and V^IV^ analogues of MFU-4l have been reported.^84,85^ With a similar ion metathesis approach, Powers has recently shown that a MOF can be used to guide the formation of metastable Pd_2_ tetracarboxylate entities.^86^ Pd(ii) acetate self assembles as a trimer (*i.e.* Pd_3_(OAc)_6_) both in the solid-state and in solution but when the chemistry is templated by existing MOF nodes these metastable Pd_2_ tetracarboxylate species can be prepared. While these approaches all provide access to reactive metal sites not observed in analogous molecular systems, the metal loading across these high symmetry sites is insufficient for structure determination by X-ray crystallography.

As noted, the formation of coordinatively unsaturated nodes for reversible small molecule binding (*e.g.* gas adsorption) is a topic of some interest. Using a desolvated form of Fe-based MOF-74 (CPO-27, Fe_2_(dobdc)_3_ (dobdc = 2,5-dihydroxy-1,4-benzene dicarboxylate)) Long and co-workers^79^ studied oxygen reactivity at the isolated coordinatively unsaturated metal node in the infinite Fe node. MOF-74-Fe exhibits temperature dependent oxygen uptake properties; below 211 K oxygen adsorption is reversible but above this temperature the process becomes irreversible. Spectroscopic data are consistent with partial charge transfer from Fe^2+^ to oxygen at low temperature and complete charge transfer at room temperature (*i.e.* Fe^3+^–O_2_^2−^). Structure insight was achieved through Rietveld analyses of powder neutron diffraction data (4 K) showing oxygen bound in a symmetric side-on mode and in a slipped side-on mode when oxidized at room temperature.

Another method for isolating catalytically active and hence reactive species at the MOF nodes is to chemically append molecular species *via* direct coordination. Lin *et al.* reported that deprotonation of the Zr_3_(μ_3_-OH) sites in UiO-68 (UniversitetIt i Oslo) with *n*-BuLi to access Co- or Fe-functionalised UiO materials (UiO-CoCl and UiO-FeBr).^87^ Compositional analysis by ICP-MS and TEM confirmed installation of the post-synthetic metal species but, again due to the high symmetry of the site, SCXRD could not provide definitive structural elucidation. However, the coordination environments were examined by XAS which suggested that in UiO-CoCl and UiO-FeBr the Co and Fe centres were coordinated to three oxygen atoms of the node and one halogen atom. Using this strategy, the same research team examined reactive magnesium alkyl catalysts using a UiO-69-NO_2_ platform but for the same reasons structural insight could not be garnered.^88^

Hupp, Farha and co-workers have used NU-1000 (Northwestern University) as a platform to generate catalysts by attachment of coordinating groups to the Zr node, followed by installation of the metal centre.^89^ A Ni^2+^-metalated form, NU-1000-(bpy)Ni^2+^, was prepared by solvent-assisted ligand incorporation of 2,2′-bipyridine to the Zr_6_-metal node which could be post-synthetically metalated with NiCl_2_ to give a “(bpy)NiCl_2_” moiety. Treatment of NU-1000-(bpy)Ni^II^ with Et_2_AlCl affords a single-site catalyst with excellent catalytic activity for ethylene dimerization and stability. However, like other examples of attachments at metal node, characterisation represents a considerable challenge. For example, the added 2,2′-bipyridine ligand is attached to an open, high symmetry crystallographic site, with only partial loading, and *via* a flexible saturated CH_2_ group. Furthermore, the Ni^2+^ centre is then attached to this site, albeit with near quantitative loading. These features prevent SCXRD for this node-appended system and more generally for this type of matrix isolated metal species.

Atomic layer deposition (ALD) has also been shown to be an effective strategy for grafting reactive sites to MOF nodes. This approach is particularly advantageous for forming reactive metal centres as the reaction conditions can be precisely controlled, and water can be specifically excluded unless required.^90,91^ ALD was used to form an efficient gas-phase hydrogenation catalyst in NU-1000 by introducing coordinatively unsaturated Ni atoms at the metal node to form NU-1000-Ni.^92^ The chemical composition of NU-1000-Ni was established but, likely due to the harsh conditions needed for ALD and the high symmetry of these sites, structural insight *via* X-ray crystallography was not reported. Structural insight was garnered from EXAFS and XANES data, complemented by DFT calculations. Using a similar approach, nickel sulfide,^93^ niobium oxide^94^ and cobalt oxide doped NU-1000 catalysts^95^ have also been prepared. A suite of techniques was employed to characterise these node-appended metal complexes, including analysing differences in the surface electron envelope between a post-synthetically metalated MOF and the parent material. In these examples,^94,95^ the difference envelope densities (DEDs) of metalated MOF samples were used to indicate the general position of the added metal ions. Reactive homogenous organometallic species can also be anchored and studied in this way, with the Hf-based analogue of NU-1000 to support a highly electrophilic single-site d^0^ Zr–benzyl complex.^96^ Again, a suite of spectroscopic techniques coupled with DFT modelling had to be relied upon to provide a putative structure of Zr–benzyl complex appended to the MOF as the high symmetry prevents SCXRD being used.

In summary, the rich coordination chemistry and the site isolation afforded at the metal nodes of MOFs can support a range of reactive species. However, the high symmetry of these sites and the challenges in achieving quantitative metal exchange or addition often precludes SCXRD. This has spurred the use of a battery of techniques to characterise such species, thereby uncovering unique chemical environments for metal centres and unusual reactivity. In Section 5 we discuss how such sites could be made amenable to structure determination by SCXRD.

### (b) MOF linker matrix isolation

Tethering metal complexes can also be achieved through suitable functionalisation of the bridging ligands that generate the extended framework structure. A representative example of this approach is to employ a dicarboxylate ligand that contains a suitable binding site positioned along the body of the ligand, such as 2,2′-bipyridine-5,5′-dicarboxylate. Many variations on this strategy can be envisaged, providing multiple pathways to the introduction of reactive metal complexes in either a pre-metalation or post-metalation manner. As the metal centre is typically tethered at a lower symmetry site in this approach, structure determination by SCXRD becomes more accessible. This is sometimes aided by the use of flexible linker sites, and having a high level of metal loading at the site of interest.

#### Pre and *in situ* linker metalation

Introducing a reactive species *via* pre-metalation requires the synthesis of a metal complex that contains additional metal binding sites exo to the complex, providing divergent binding modes needed for MOF formation. For example, using 2,2′-bipyridine-5,5′-dicarboxylic acid, or 2,2′-bipyridine-4,4′-dicarboxylic acid, as a ligand allows formation of a complex in which the bipyridine site is used to tether the target complex leaving the carboxylic acid groups unreacted for subsequent MOF formation. This approach has been demonstrated for a range of targets^97,98^ and has been used to successfully incorporate [M(bipy)_3_] complexes,^99–101^ [M(bipy)_2_(CN)_2_]^101,102^ [M = Ru(ii), Os(ii)], [Ir(bipy)(Cp*)Cl],^103^ [Ir(bipy)Cl_3_(THF)],^104^ or 2-phenylpyridine (ppy) complexes, such as [Ir(ppy)_2_(bpy)]^+^ (ref. 105) [Ir(Cp*)(ppy)].^106,107^ However, depending on the MOF being prepared, it can be highly challenging to obtain single crystals of these materials despite the enforced high occupancy of the pre-metalated complex.

**Fig. 4:**
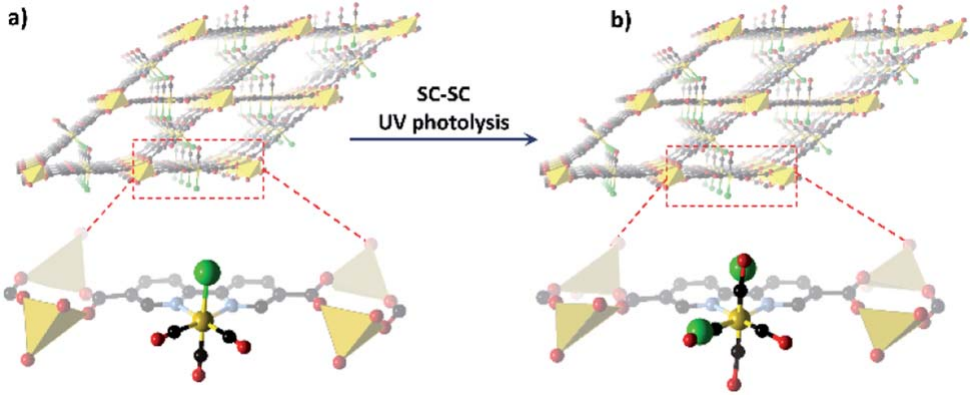
(a) View of MOF formed by {Mn(DMF)(OH_2_)[(2,2′-bipyridine-5,5′-dicarboxylate)Mn(CO)_3_Cl]}_*n*_, noting the *fac*-M(diimine)(CO)_3_X moieties tethered to the framework structure.^15^ Upon UV photolysis, a portion of the Mn(i) centres adopt the *mer* geometry (b), as revealed by X-ray crystallography (Mn – beige; O – red; C – black, N – lavender, –Cl – green).

A representative system that has allowed the growth of single crystals and subsequent study of the tethered complex's reactivity are those that use M(2,2′-bipyridine-5,5′-dicarboxylate)(CO)_3_X (M = Re, Mn, X = Cl, Br) as a ligand in the construction of MOFs.^70,108–110^ In this series of compounds the carboxylate donors of the 2,2′-bipyridine-5,5′-dicarboxylate ligand binds Mn(ii),^70,108^ Li(i)^109^ or Cu(ii)^110^ to generate MOFs (Fig. 4). Interestingly, all three MOFs possess superficially similar framework structures with an analogous arrangement of the M(diimine)(CO)_3_X moieties, despite the frameworks having distinct topologies.^70,109,110^ These systems are of interest as the photochemical behavior of M(diimine)(CO)_3_X complexes has been studied extensively. Thus, these tethered complexes provided an ideal platform to investigate the influence of the isolating MOF environment on photochemical behaviour using time-resolved infra-red (TRIR) spectroscopic studies. In the case of the MOFs that employ Mn(ii) as a MOF-node ({Mn(DMF)_2_[(2,2′-bipyridine-5,5′-dicarboxylate)Re(CO)_3_X]}_*n*_ or ({Mn(DMF)_2_[(2,2′-bipyridine-5,5′-dicarboxylate)Mn(CO)_3_X]}_*n*_; X = Cl, Br) the TRIR studies confirm the formation of both ^3^MLCT and ^3^IL (intraligand) π–π* states. The ^3^MLCT bands are unstable, decaying rapidly (*ca.* 20 ps) with concurrent further growth of the intraligand^3^ π–π* 1 ns after laser excitation only the intra-ligand^3^ π–π* states are present. In solution the ^3^MLCT state is normally observed for Re(bipy)(CO)_3_Cl species and the higher energy^3^ π–π* state is not normally accessible. Therefore, it was concluded that the MOF environment induces a change in the nature of the excited states of the framework-supported complex. For both {Mn(DMF)_2_[(2,2′-bipyridine-5,5′-dicarboxylate)Re(CO)_3_Cl]}_*n*_ and {Mn(DMF)_2_[(2,2′-bipyridine-5,5′-dicarboxylate)Mn(CO)_3_Cl]}_*n*_, irradiation for 22 hours at 200 K leads to the observation of free CO in the MOF. If such samples are warmed above 250 K formation of the corresponding *mer*-isomer of the M(diimine)(CO)_3_X species, from the initial *fac*-isomer, is observed. In the case of {Mn(DMF)_2_[(2,2′-bipyridine-5,5′-dicarboxylate)Mn(CO)_3_Cl]}_*n*_, 25% conversion from *fac*- to *mer*-isomer is observed allowing characterisation of the photoinduced isomerisation products by SCXRD.

Variation of the metal cations that lead to MOF propagation, from Mn(ii)^70,108^ to Li(i)^109^ or Cu(ii),^110^ results in modification of the photophysical behavior of the tethered M(diimine)(CO)_3_X moiety. In the case of {Li(DMF)_2_[(2,2′-bipyridine-5,5′-dicarboxylate)Re(CO)_3_Cl]}_*n*_ luminescence is observed and the excited state behaviour,^109^ established using TRIR measurements, resembles that of unsubstituted [Re(bipy)(CO)_3_Cl] rather than that observed for {Mn(DMF)_2_[(2,2′-bipyridine-5,5′-dicarboxylate)Re(CO)_3_Cl]}_*n*_, discussed above. In the case of {Cu(DMF)(OH_2_)[(2,2′-bipyridine-5,5′-dicarboxylate)Re(CO)_3_Cl]}_*n*_ TRIR spectroscopy reveals that prolonged irradiation of samples leads to the irreversible formation of a low quantum yield photoproduct, potentially as a result of photoinduced charge transfer followed by decomposition.^110^

Using a similar strategy, structural characterisation of pre-metalated N-heterocyclic carbene (NHC) complexes was also achieved by single-crystal X-ray diffraction.^111^ The structure reveals that IRMOF-77 (which contains an NHC–PdI_2_(py) moiety) is isoreticular with MOF-5 although, due to the larger linker, interpenetration is observed. The presence of the NHC–PdI_2_(py) moiety, which lacks the steric bulk normally needed for such NHC species to be stable in solution, was confirmed. Due to the material being porous, the Pd site is available for further chemistry; however, these transformations did not occur with retention of the single crystalline nature of the sample.

A related approach to include reactive organometallic complexes was pursued by Humphrey *et al.*^112,113^ Seeking to achieve air and moisture stability, the researchers used a tetra(carboxylated) P–C–P pincer ligand to target highly connected, robust MOFs. Cyclometalation of the P–C–P pincer with Pd^2+^ gave a metalloligand which could be used to prepare a crystalline 3D MOF (PCM-36, Phosphorus Coordination Material) with Co^2+^ ions. Structural determination by SCXRD showed the (P–C–P pincer) Pd–Cl groups inside the pores are accessible to post-synthetic modification, including formation of a Pd–CH_3_ alkyl complex that undergoes rapid insertion of CO_2_ gas (1 atm and 298 K) to give Pd–OC(O)CH_3_. Due to the CO_2_/CO adsorption preferences of the MOF, reaction outcomes can be controlled and a Pd–N_3_ species is resistant to CO insertion under the same conditions. While the initial compound was amenable to SCXRD, subsequent chemistry did not occur with retention of single crystallinity. A similar strategy using P–N–P pincer ligands was followed by Wade to introduce Ru centres into a MOF as a hydrosilylation catalyst.^114^ Reaction of the Ru pincer metallolinkers with ZrCl_4_ gave three MOFs (Zr_6_O_4_(OH)_4_(OAc)_4_{*cis*-(P^N^N^N^P)RuCl_2_(CO)}_2_, Zr_6_O_4_(OH)_4_(O_2_CH)_4_{(P^N^N^N^P)RuCl(CO)_2_}_2_Cl_2_, and, Zr_6_O_4_(OH)_4_(OAc)_4_{*cis*-/*trans*-(P^N^N^N^P)RuCl_2_(CO)}_2_; P^N^N^N^P = 2,6-(HNPAr_2_)_2_C_5_H_3_N; Ar = *p*-C_6_H_4_CO_2_^−^) with subtly different coordination environments at Ru. While SCXRD was not possible, a suite of techniques, including synchrotron X-ray powder diffraction, provide the structure of the P–N–P–Ru pincer MOFs. Zr_6_O_4_(OH)_4_(O_2_CH)_4_{(P^N^N^N^P)RuCl(CO)_2_}_2_Cl_2_ was able to be treated over two steps with KO^*t*^Bu to deprotonate the NH group of the P–N–P–RuCl(CO)_2_ linker and Me_3_NO to remove a Ru-coordinated CO ligand. The product, which was characterised spectroscopically, proved to be a recyclable catalyst for the hydrosilylation of aryl aldehydes with Et_3_SiH, outperforming an equivalent homogeneous analogue.

Shifting from a pre-metalation to an *in situ* metalation strategy similarly provides materials that can isolate transition metal phosphine complexes and facilitate their structural characterisation. Specifically, a one pot, *in situ* formation of bimetallic MOF was demonstrated where low-valent phosphine metal complexes could be crystallographically characterised.^115^ The concomitant complexation of the phosphorus centre with a low valent metal and MOF formation *via* a metal-carboxylate network with a high valent node occurs without interference, to afford two new phosphine coordination materials, PCM-107 and PCM-74. These materials feature Ar_3_P–AuX (X = Cl, Br) and chelated Cu_2_I_2_ units (P–N coordinated) in their final structures. Interestingly PCM-74 traps a kinetically favoured isomer of the Cu(i) complex but in both cases, and despite PCM-107 being an active catalyst for alkyne hydroaddition, no structural insights into further reactivity could be garnered.

One clear advantage of the pre-metalation approach is that the metal reaction site and its reactivity can be tuned by the formation of different MOFs, as shown for the photochemistry of M(diimine)(CO)_3_X moieties. A common theme in this work, and other studies in this section, is that the MOFs are commonly formed with low valent, labile metal nodes which facilitate facile crystallization of the MOF. However, retaining open and permanently porous networks that facilitate access to the reactive metal species can be more challenging, as MOF synthesis needs to consider the stability of the pre-metalated complex. An example, reported by Wade,^114^ using high valent node metals could not be characterised by SCXRD but did enable multi-step transformations on the tethered metal centre without loss of structure. In summary; however, with the caveat that the complex-bearing ligand must be stable with respect to the conditions of the MOF formation reaction, the pre-metalation approach to making MOFs containing tethered metal complexes is a highly efficient and adaptable strategy.

#### Post-synthetic linker metalation

Since many transition metal complexes are incompatible with solvothermal MOF synthesis conditions, post-synthetic metalation (PSMet)^30^ of linker sites provides an important pathway towards the heterogenisation and structural characterisation of reactive metal species. An early example of matrix isolation in a MOF *via* linker binding involved PSMet of the arene rings of MOF-5 with organometallic Cr, Mo and W tricarbonyl complexes.^116–118^ The choice of this chemistry alleviates the challenges of retaining free coordinating sites, as the arene rings are a ubiquitous presence in MOF linkers. The reaction between MOF-5 and [Cr(CO)_6_] in dibutyl ether and THF at 140 °C afforded quantitative formation of the corresponding η^6^-arene tricarbonyl complex.^116^ However, given rotational freedom of the linker, SCXRD was not able to provide structural characterisation, but it was notable that 450 nm photolysis under an N_2_ or H_2_ flow generates the corresponding η^6^-arene-[Cr(CO)_2_L] complex (L = N_2_ or H_2_). Site isolation within the MOF matrix substantially extends the lifetime of the arene–[Cr(CO)_2_H_2_] complex compared to the molecular analogue, further substantiating the premise that MOFs can serve as crystalline matrices for the isolation of reactive species.

**Fig. 5:**
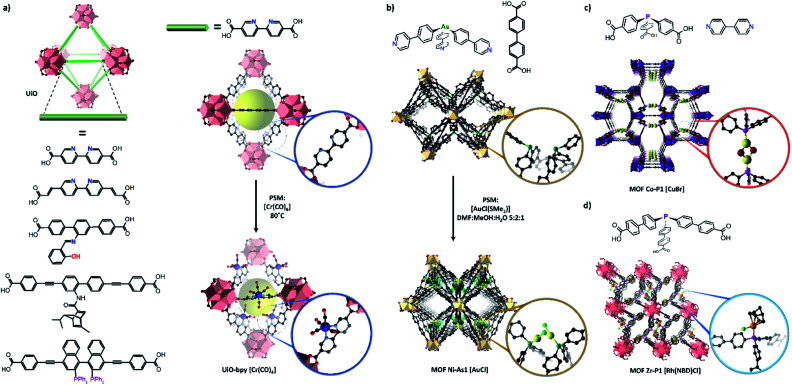
MOFs bearing diverse free-coordination sites have been prepared for post-synthetic metalation, including numerous derivatives of the UiO structure (a). UiO frameworks based on the short, relatively rigid 2,2′-bipyridine dicarboxylate linkers have been structurally characterised following metalation with transition metal complexes including Cr(CO)_6_. Other MOF materials bearing organoarsine (b) and phosphine (c and d)) coordination sites have also been structurally characterised following metalation, allowing determination of the structures of the added metal centre (Zr – salmon pink; O – red; C – black, Ni – beige, Co – purple, Au – yellow, Cl – green, Rh – orange, Cu – yellow, Br – maroon, P – pink, As – dark green, Cr – dark blue).

The study of other reactive species or catalysts requires the use of linkers featuring specialised binding sites such as N, O or P donors, which must remain available in the MOF for PSMet. Strategies to effect this were comprehensively reviewed.^30^ Consequently, one of the most expansive platforms for PSM chemistry is the UiO-type frameworks which can be prepared from a broad repertoire of functionalised, linear dicarboxylate-linkers.^119^ UiO-derivatives featuring diene,^120^*N*,*N*-chelate,^121,122^ salicylaldimine,^123^ diol,^124^ BINAP,^125^ NHC^126^ and β-diketimine^127^-based donors (Fig. 5) have been metalated to yield site-isolated metal complexes with enhanced stability compared to their homogeneous counterparts.^97,120,122,128,129^ In selected cases, on which we will focus, post-synthetically installed transition metal complexes have been structurally characterised in UiO-67-bpy (where bpy = 2,2′-bipyridine-5,5′-dicarboxylate);^128,130,131^ however, these examples remain scarce and the material is more extensively used as a stable platform material. This is mainly because UiO materials do not readily form single crystals and, furthermore, possess a high density of metalation sites in a rigid high symmetry framework, features which make SCXRD more challenging.

Site-isolation of ligand binding sites in UiO-derivatives, which can be enhanced by mixed-linker approaches (to the detriment of structure determination by SCXRD), has been employed to prepare novel catalysts that negate the dimerization and ligand disproportionation reactions that encumber such species in solution.^120^ In a salient study, Lin *et al.* metalated UiO-67-bpy and UiO-67-phen (where phen = 1,10-phenanthroline-3,8-dicarboxylate) derivatives with CoCl_2_. Using NaB(Et)_3_H the Co(ii) centre was reduced to form the corresponding UiO-67-ligand(*N*,*N*)-[Co(THF)_2_] complex which is a powerful hydrogenation catalyst.^132^ In solution, the equivalent reaction leads to ligand disproportionation and nanoparticle formation, reflecting the value of the MOF as a platform for isolating highly active, otherwise inaccessible catalysts. While matrix isolation of reactive species was clearly demonstrated, garnering definitive structural insight into these complexes by SCXRD was not possible. Other non-linear links can be used to prepare Zr-based MOFs featuring bespoke coordination sites.^133,134^ This deviation from linearity can lower the crystallographic symmetry of the metal coordinating site and favour structure determination.

Dincă *et al.* employed a novel strategy to incorporate a ‘scorpionate’-type *N*,*N*,*N*-chelation site into a Zr framework.^135^ The flexibility of the ligand, *tris*-(4-carboxy-3,5-dimethylpyrazol-1-yl)methane, precluded direct synthesis of a Zr based framework. Instead, the *N*,*N*,*N*-chelation site was metalated with CuI during MOF synthesis, creating a rigid entity that readily forms the desired Zr based MOF. The CuI can be removed by treatment with acid, generating a chelating site for post-synthetic metalation with the more reactive [Cu(NCCH_3_)_4_]BF_4_. Another tripodal ligand, 4′,4′′′,4′′′′′-phosphanetriyltris([1,1′-biphenyl]-4-carboxylic acid), has been used by Lin *et al.* to prepare a Zr(iv) based MOF Zr–P1 featuring free phosphine donors which undergo post-synthetic metalation with Rh(i)^136^ and Ir(i)^134,136^ complexes. Phosphines are featured extensively in homogeneous catalysts due to their strong and tuneable coordinating ability; however, homogeneous systems can suffer from ligand disproportionation and nanoparticle formation. MOF-Zr-P1 acts as a platform for accessing supported monophosphine Rh and Ir catalysts, negating the need for sterically bulky phosphine ligands that can reduce catalytic activity. The reaction between MOF-Zr-P1 and [Rh(NBD)Cl]_2_ or [Ir(COD)OMe]_2_ yields the corresponding MOF-Zr-P1[Rh(NBD)Cl] (Zr–P1–Rh) or MOF-Zr-P1[Ir(COD)OMe] (Zr–P1–Ir) complexes respectively, which was verified by EXAFS and by X-ray crystallography in the case of Zr–P1–Rh. PSM is critical due to the sensitivity of the Rh(i) and Ir(i) precursors and such low coordinate Ir(i) complexes are not accessible in solution due to the formation of metallic particles and ligand disproportionation. Although while an initial structure could be characterised by X-ray crystallography for a single example, structural insights by SCXRD into the reaction chemistry were not possible.

Seminal works by Humphrey *et al.* described the preparation of mixed-linker MOFs featuring free phosphine^137^ or arsine^138^ donor sites which are accessible *via* porous channels. The monodentate P- or As-donors undergo PSM with retention of framework crystallinity, allowing the new metal centres to be studied *via* X-ray crystallography. In the arsine material, the free As sites of two adjacent linkers are spatially arranged in a *cis*-fashion and separated by 4.8 Å. PSM with AuCl(Me_2_S) yields the corresponding As(AuCl) complexes which are accommodated by a distortion of the MOF framework. Due to the rigid structure of the MOF host and proximity of the arsine sites, the Au centres feature an unusually short contact distance of 2.76 Å. The phosphine based material also features proximate donor sites, but arranged in a trans fashion and separated by 7.22 Å, thus creating a *P*,*P* chelation site. Post-synthetic metalation with AuCl(Me_2_S) or CuBr(Me_2_S) yielded the corresponding P(AuCl) complex and chelated dimer *P*,*P*(CuBr)_2_, which were still able characterised using X-ray crystallography despite respective site occupancies of 63 and 53% determined by ICP-OES.

By incorporating structural flexibility into the MOF design, the host framework can accommodate the structural changes required for post-synthetic modification without losing long-range order. Li and Zhou *et al.* reported a Zr-based framework, HUST-1 (Huazhong University of Science and Technology), in which *N*,*N* chelating sites are generated by the judicial arrangement of flexible 4,4′-(4-amino-4*H*-1,2,4-triazole-3,5-diyl)phenyl-carboxylate ligands bearing monodentate triazole sites.^139^ The adjacent triazole pairs provide chelating sites for metal clusters. PSM with [Ni(OH_2_)_6_]Cl_2_ or [Ni(OH_2_)_6_]Br_2_ yields hepta-nuclear or tri-nuclear Ni clusters respectively, which are bound to the *N*,*N* chelation site formed between adjacent linkers. The flexibility of the MOF appears to allow the material to accommodate metalation without losing crystallinity, thereby facilitating the structural characterisation of the Ni clusters *via* X-ray crystallography.

In the few cases where SCXRD of metalated UiO-67-bpy materials is possible, successful structural characterisation seems to require distortion of the local structure, lowering symmetry and limiting rotational movement that may give rise to disorder.^128,130,133,140^ These features were observed for the first characterisation of metalated UiO-67-bpy derivatives,^128,130^ and are observed in the latest contribution by Long *et al.*^131^ In the latter study^131^ large metal halide sheets (MX_2_ where M = Ni, Co, Fe, and X = Cl and Br) could be isolated and characterised by SCXRD within the pores of UiO-67-bpy. Structure determination shows that the metal halide sheets are effectively excised from the bulk metal halide structure but terminated and stabilised by peripheral bipyridine coordinating groups. Remarkably, the steps leading to assembly metal halide sheets can be studied by SCXRD structures obtained at different metal loadings, providing insight into the growth mechanisms.

**Fig. 6:**
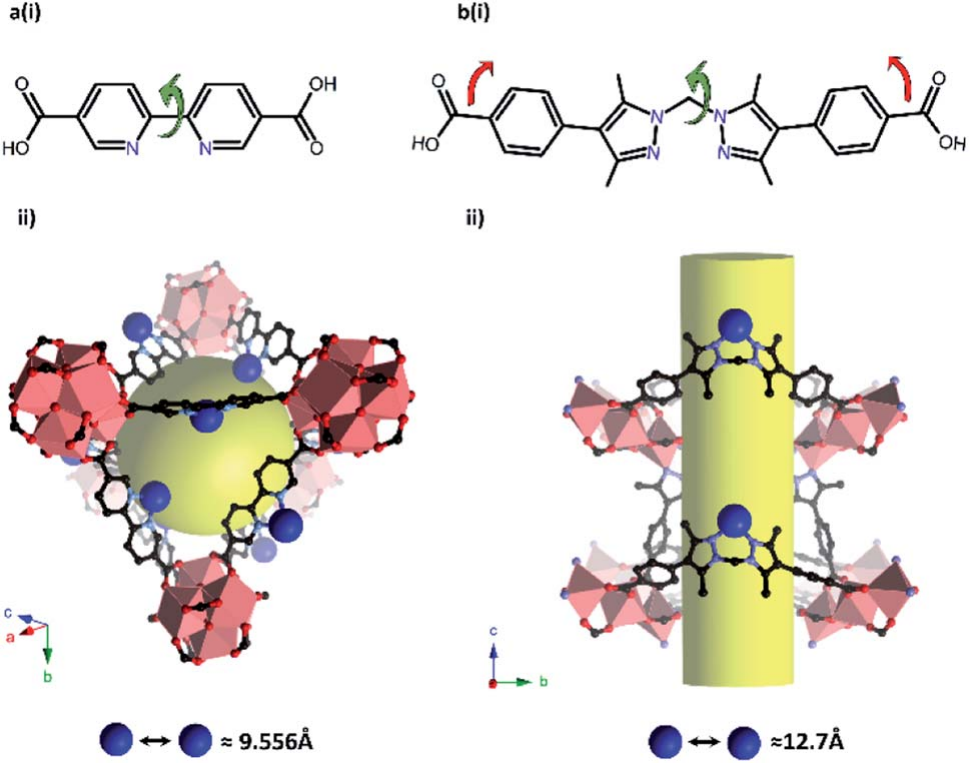
Ligands bearing *N*,*N*-chelation sites used to prepare UiO-67-bpy (a) and MnMOF-1 (b). Flexibility (red arrows) and rotational freedom (green arrows) exhibited by the MnMOF-1 linker result in a flexible MOF structure. In addition, the arrangement of free coordination sites, and therefore chelated metal centres (blue spheres) in UiO-67-bpy (a(ii)) is more dense than in MnMOF-1 (b(ii)). Although this can be alleviated using extended linkers in the UiO system, such MOFs do not lend themselves to structural elucidation following metalation (MOF nodes – salmon pink polyhedra; O – red; C – black, N – lavender, metalated site – blue).

Most examples presented thus far in this section demonstrate isolation and structural characterisation of the initial metalation product. Clearly, a key advantage of incorporating reactive species, such as catalysts, within a porous, crystalline MOF matrix is the prospect of obtaining structural insight not just into the initial state of the catalyst, but also its evolution during catalysis. Recent advances have realised this potential, most notably using the Mn-derived framework MnMOF-1 ([Mn_3_(L)_2_L′], where L = bis(4-carboxyphenyl-3,5-dimethyl-pyrazol-1-yl)methane).^141,142^ Compared to UiO-67-bpy, this framework exhibits particular qualities as a host for post-synthetic metalation chemistry (Fig. 6). MnMOF-1 consists of Mn(ii) trimers which are interconnected by L moieties, designated L′, that coordinate exclusively *via* their carboxylate donors, leaving the non-linear *N*,*N*-chelation site free to coordinate to guest metal complexes. The flexibility provided by the ligand, compared to 2,2′-bipyridine derived links (Fig. 6), appears to allow the material to adapt structurally to accommodate both the initial metalation, and the changes in the coordination sphere of the included metal centre, without compromising the crystallinity of the host framework. Furthermore, the lower symmetry space group of MnMOF-1, combined with the non-linear *N*,*N*-chelation site (compared to 2,2′-bipyridine again) renders the axial sites in coordinated complexes crystallographically independent and aids the structural elucidation of the included compounds. As a result, the material acts as a crystalline matrix within which coordination chemistry can be studied in exemplary detail.^142–145^

**Fig. 7:**
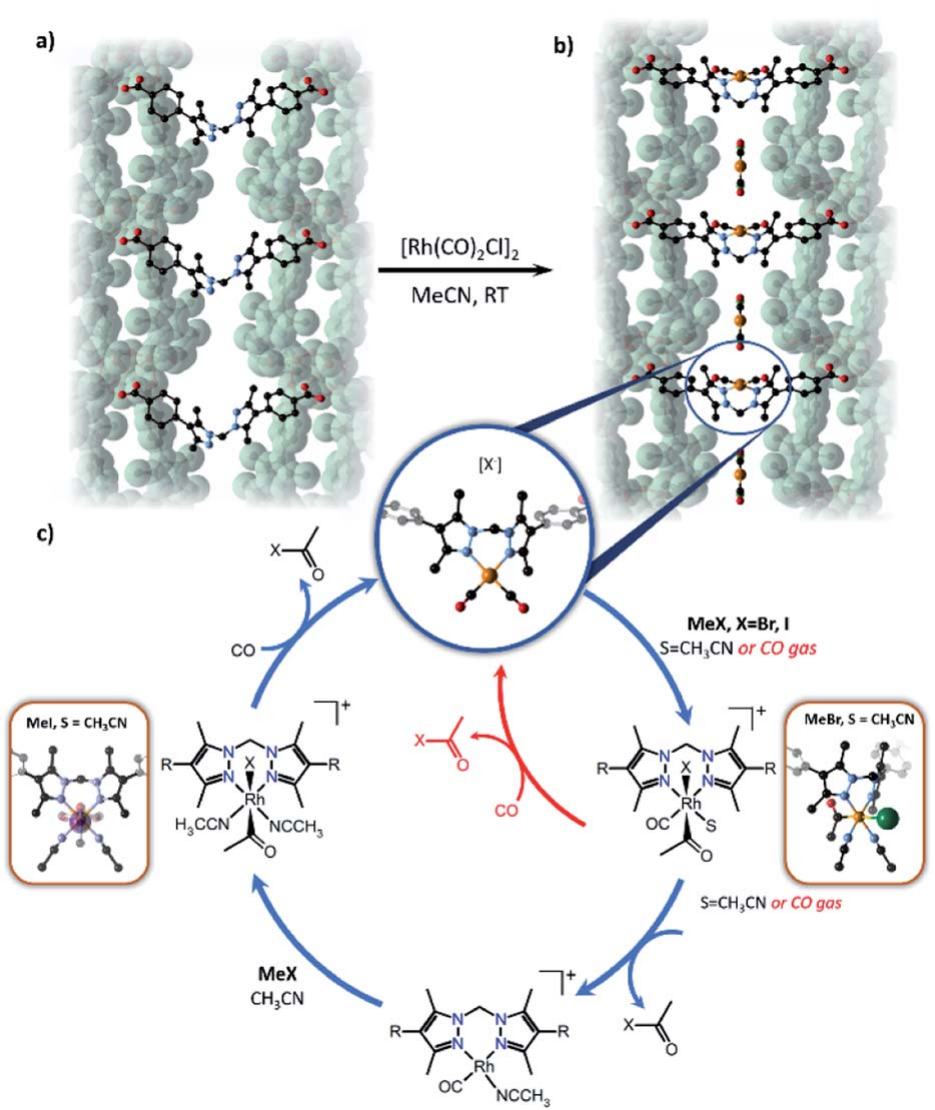
Metalation of MnMOF-1 (a) with [Rh(CO)_2_Cl]_2_ yields MnMOF-1 [Rh(CO)_2_][Rh(CO)_2_Cl_2_] (b). The proposed catalytic cycle for the carbonylation of methyl iodide using the Rh(i) functionalised MOF is depicted (c) for both the gas phase (red) and solution phase (blue). R = MOF framework (MOF backbone – green surface, C – black, N – lavender, O – red, Rh – orange, I – green).

Reactions involving the octahedral-tetrahedral interconversion of Co(ii) complexes^141^ and sequential anion exchange and [2 + 3]-cycloaddition chemistry of a Mn(i) complex^145^ have all been studied inside MnMOF-1 using X-ray crystallography. Furthermore, studying reactivity using MnMOF-1 has recently been extended to organometallic catalysis.^142^ The Monsanto acetic acid synthesis is an important industrial chemical processes, performed using homogenous Rh(i) carbonyl catalysts.^146^ The mechanism involves oxidative addition of MeI to the metal centre, migratory insertion of the methyl fragment into the M–CO bond and reductive elimination of acetyl iodide to regenerate the square planar catalyst. To demonstrate that MOFs could provide structural insight into such varied chemical reactivity, MnMOF-1 was metalated with an organometallic Rh(i) complex, forming MnMOF-1[Rh(CO)_2_][Rh(CO)_2_Cl_2_] (Fig. 7). The structure was determined using X-ray crystallography, which confirmed the presence of the square planar Rh(i) species and charge-balancing [Rh(CO)_2_Cl_2_]^−^ counterion. Notable stability of the Rh(i) compound was achieved due to incorporation in the MOF.^141^ The Rh(i) metalated material reacts with MeI in acetonitrile to give red-orange crystals suitable for X-ray crystallography; structure determination revealed the presence of the octahedral Rh(iii) oxidative addition product **1**·[Rh(CO)(CH_3_CO)I(MeCN)]^+^. Intriguingly, the reaction with MeBr produces the oxidative addition product **1**·[Rh(CH_3_CO)Br(MeCN)_2_]·Br in which the metal centre has undergone reductive elimination of acetyl bromide and a second oxidative addition, thereby consuming both CO ligands. The disparity between the structures obtained from MeI and MeBr oxidative addition are attributed to the steric demands of the respective halide ligands. Following oxidative addition to give the halide complex, the acetyl and halide ligands reside on the axial sites of the Rh complex and must rearrange to give the *cis* complex in order for the reductive elimination of acetyl halide to occur. The smaller bromide anion readily undergoes this rearrangement, while the sterically demanding iodide cannot rearrange to form the *cis* complex and is therefore trapped after the initial oxidative addition step. These insights were crucial to explaining the catalytic activity of the Rh complex within MnMOF-1. In the gas phase, the material catalyses the production of acetyl bromide, albeit at a very slow rate, likely due to the initial oxidative addition step being significantly slower for MeBr than MeI. Although MeI undergoes more rapid oxidative addition, the sterically demanding iodine substituent fails to undergo the rearrangement to the *cis* isomer to release CH_3_(CO)I *via* reductive elimination, confirmed by the direct observation of [Rh(CO)(CH_3_CO)I(MeCN)]^+^ in the crystal structure. The smaller MeBr undergoes a much slower oxidative addition, but the smaller bromide facilitates the necessary rearrangement to release CH_3_(CO)Br. The difference in reactivity between MeI and MeBr can thereby be rationalised based on the structural insights garnered from SCXRD, particularly the observation of key intermediates in the catalytic cycle (Fig. 7). These results demonstrate the importance of incorporating a degree of flexibility into MOF scaffolds for matrix isolation; not only can the starting complex be characterised but complete reaction cycles can be followed by SCXRD.

Further success in this direction was shown by studies in metallo-porphyrin containing MOFs.^147^ PCN-224, which is a Zr-based MOF that incorporates a free tetracarboxyphenylporphyrin bridging ligand, can be subsequently metalated to introduce Fe(ii) cations into the porphyrin binding site. The Fe-containing MOF reacts with oxygen gas and the structure of the product can be confirmed by SCXRD to unequivocally establish the structure of the Fe–porphyrin O_2_ complex. Structural observation of this five-coordinate heme–O_2_ adduct, lacking an additional base, such as imidazole, is significant as such chemical species are only observed transiently in their molecular form. This study further demonstrates the advantages of not only obtaining the structural insight but of having these groups anchored and spatially isolated within the framework structure. The initial work with Fe was extended to Co and Mn derivatives to give a five-coordinate Co(iii) superoxo species^148^ and a side-on peroxomanganese(iv) species,^149^ respectively.

### (c) Matrix isolation within MOF pores.

The confining pore environment of MOFs, where additional functionality attached to the framework structure can be used to modify the size, shape and chemistry of the pore space, can also be used to stabilise reactive metal species. Some of the initial uses of MOFs as a crystallisation matrix have demonstrated this point with electron rich guests being organised and characterised by X-ray crystallography in the pores of crystalline sponge hosts.^34,39,150–155^ More recently, this concept has been extended to reactive metal-based species by installing a reactive cartridge into the pore network.^20^ Finally, inspired by the importance of metal nanoclusters (MNCs) in catalysis, a number of reports have now shown the precise structural characterisation of MNCs. These three activities all rely upon judicious selection of MOF materials possessing pore networks that can locate and stabilise the reactive metal species through interactions between the MOF pore surface and the metal species; these positioning effects are needed to achieve the long range order and the high site occupancies required for characterisation by SCXRD.^34,150^ Examples of each category are given in the following section.

While clathrate lattices formed from porphyrins have been used to structurally characterise guests contained in a lattice,^156,157^ Fujita was one of the first to advance the concept that an extended porous network material, such as a MOF, could be used as a matrix for ordering guest molecules prior to SCXRD.^34,39,150^ The particular advance achieved in the so-called crystalline sponge method removed the need to grow single crystals of a target compound. While the ability to crystallographically characterise gaseous and solvate species trapped within network of porous crystalline materials was widely appreciated, the key innovation was that any suitable new guests could be ordered within the so-called crystalline sponge to facilitate structure elucidation.^34,36,39^ Some of the methodological challenges associated with the crystalline sponge method have been tested and alternative protocols and approaches postulated.^158–160^ Nonetheless, the crystalline sponge approach has been applied to a range of organic molecules, including examples prone to decomposition, allowing structure determination of the target by SCXRD.^161–165^ However, given the hydrophobic chemistry of the pore networks of most crystalline sponges, turning this method to the isolation and structure determination of reactive, often charged, inorganic species is challenging. A contribution by Fujita reported the crystalline sponges to facilitate HPLC separations of titanocene organometallic complexes^164^ and recently confinement effects for ferrocene have been studied within a crystalline sponge.^166^ Furthermore, the crystalline sponge method certainly serves to isolate reactive species. Buchwald and co-workers used a crystalline sponge to re-evaluate the structure of an electrophilic hypervalent iodine reagent for trifluoromethylthiolation.^167^ Through application of the method, they were able to demonstrate the formation of stable thioperoxy complexes instead of the expected benziodoxole derivatives. As the chemical and structural diversity of the crystalline sponge materials increase, it is likely that the trapping of reactive inorganic species will be more widely realised and applied.

**Fig. 8:**
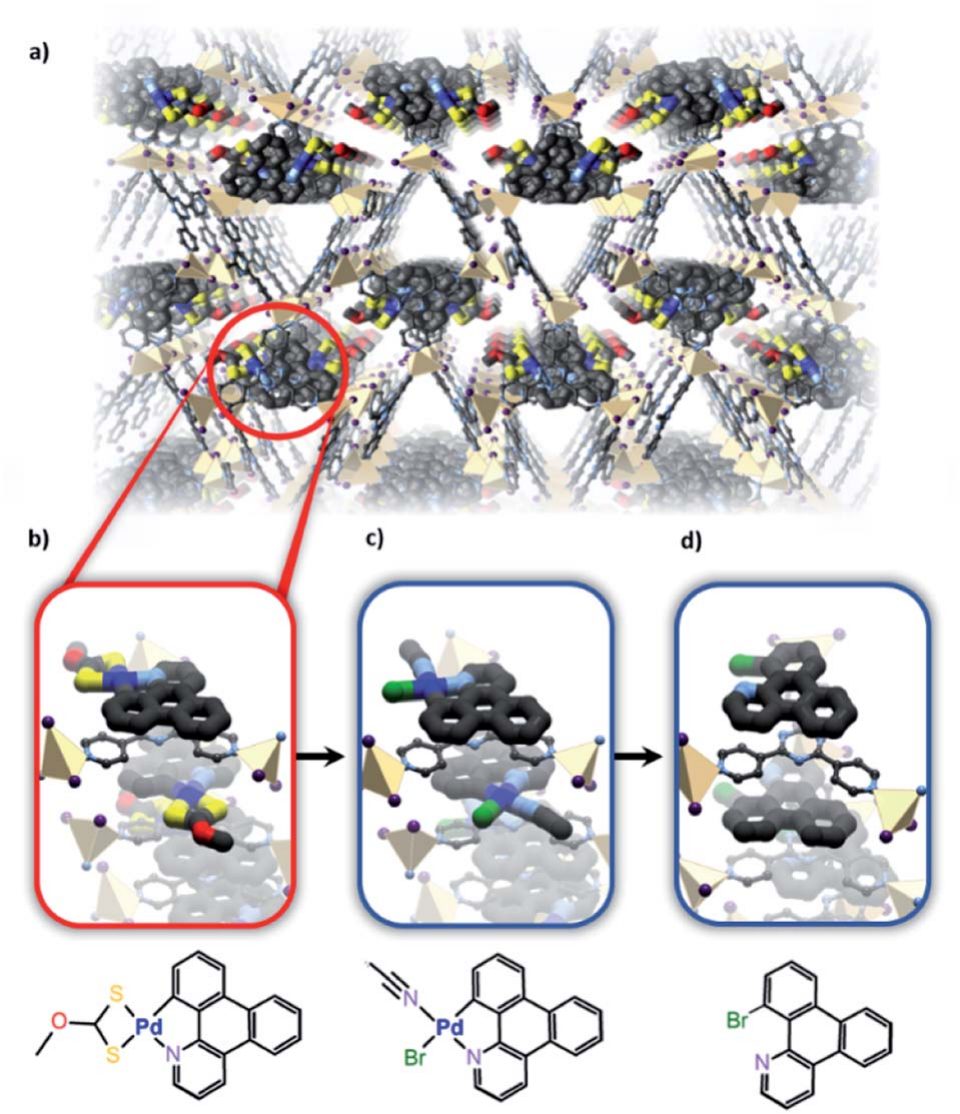
(a) The Zn based porous framework hosts the 1-azatriphenylene Pd complexes which intercalate between the *tris*(4-pyridyl)triazine ligands of the host to form infinite stacks. (b) Bromination of the initial substrate *via* reaction with NBS forms a Pd(ii) bromide complex (c) and finally yields the brominated product (d) as observed *via* X-ray crystallography (Zn – beige, S – yellow, C – black, N – lavender, O – red, Pd – blue, Br – green).

Further taking advantage of the host–guest properties of MOFs allowed Fujita and co-workers to study reactivity occurring at a cyclometallated palladium species.^20^ The researchers used a time-resolved X-ray crystallography approach to not only characterise the starting organometallic unit but also gain insight into a palladium-mediated bromination of a phenyl group (Fig. 8). Electron-density maps clearly showed the intermediacy of an Ar–Pd(ii)–Br species prior to elimination to give the aryl bromide product. The “cartridge” approach used is part of a more general approach to confer functionality to a family of MOFs and nicely demonstrates the potential for using SCXRD to follow reactions within framework materials. However, the particular requirements of the cartridge might limit the application of this approach to a wider variety of metal-centred reactions. Nonetheless, this shows how metal-based reactions can be studied in ostensibly hydrophobic materials.

Some so-called “ship in a bottle” synthetic processes and direct impregnation of metal-based catalysts have also facilitated the matrix isolation of reactive metal-based species within MOF pores. Examples include using bio-MOF-1 (Zn_8_(ad)_4_(bpdc)_6_O·2Me_2_NH_2_; ad = adeninate), as a host for synthesising Co(ii), Ni(ii) or Cu(ii) phthalocyanine (MPc) in bio-MOF-1 (*i.e.* CoPc@bio-MOF-1, NiPc@bio-MOF-1 and CuPc@bio-MOF-1),^168^ the synthesis of a Co oxime complex in NH_2_-MIL-125(Ti) (MIL = Materials Institute Lavoisier),^169^ [Rh(dppe)(COD)]BF_4_ in ZJU-28 (ZJU = Zhejiang University),^170^ or encapsulating Crabtree's catalyst inside the pores of sulfonated MIL-101(Cr).^171^ These encapsulated catalysts are often more stable, and can show remarkable activity and selectivity between products due to the sterically and chemically tuneable environment; however, due to the typically sub-stoichiometric occupancy or the high degrees of freedom provided by large MOF pores these species cannot always be directly visualised by SCXRD.

The isolation and structural characterisation of well-defined ultra-small (sub 2 nm) aggregations of metal atoms, – so-called metal nanoclusters (MNCs),^172^ has also garnered significant attention. A series of recent works have unveiled superlative catalytic activity^173–176^ for these species. Unfortunately, despite such importance, the gram-scale synthesis of structurally and electronically well-defined ligand-free MNCs still constitutes a challenge. Matrix isolating such species within the pores of a MOF may offer opportunities to facilitate (i) their synthesis and characterisation; (ii) their stabilisation without protective ligands that may reduce or block their catalytic properties, but prevent their rearrangement into MNPs and, especially, (iii) their synthesis in a multi-gram scale, enabling industrial applications.^172^ MOFs are particularly suitable for this purpose but also provide, theoretically, the possibility to use SCXRD as a powerful tool to shed light on the crystal structure of MNCs. Certainly, a number of publications reporting the encapsulation MNCs within MOFs have been published. However, in this review we have only focussed on examples where ligand-free well-defined ultrasmall MNCs are isolated and structurally characterised.

The main strategies to obtain these MOF-encapsulated MNCs can be broadly divided in two main blocks: on the one hand, MOFs could be assembled around preformed MNCs (“bottle around ship”). On the other hand, MOFs could be also used as “vessels” for the straight insertion of preformed functional guest species or used as chemical reactors for the *in situ* preparation of such ultrasmall active species (“ship in a bottle”).^177^ More specific strategies include encapsulation, *via* a one-step, single pot reaction wherein MNC growth is concomitant with MOF synthesis; a multistep process where MNCs are formed with capping ligands and followed by MOF assembly, or post-synthetic methods, for example, chemical vapour deposition (CVD) and gas-phase solid grinding. However, most of these strategies and approaches give access to well defined mixtures of nanoparticle clusters which due to their inhomogeneity of size, structure and composition are not amenable to structure determination by SCXRD. There are one or two exceptions to this observation, namely the work of Zhu *et al.*^178^ who reported a modified solvent assisted impregnation method for the preparation of atomically-precise homo- and bimetallic MNCs, specifically Au_11_:PPh_3_ and Au_13_Ag_12_:PPh_3_, within ZIF-8 and MIL-101(Cr) MOFs, respectively.

**Fig. 9:**
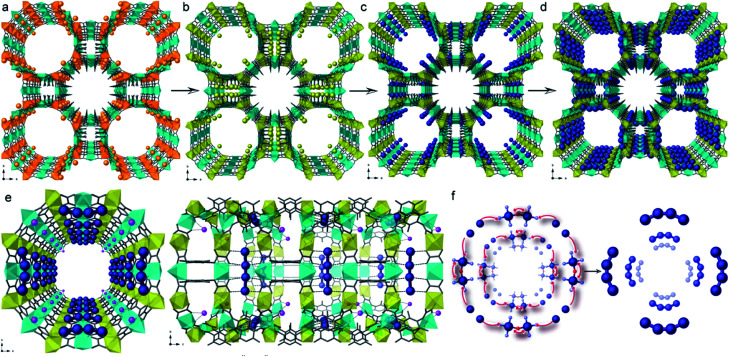
Three-step synthesis of [Pd_4_]_0.5_@Na_3_{Ni^II^_4_[Cu^II^_2_(Me_3_mpba)_2_]_3_} consisting of a transmetallation of the starting {Cu_6_Mg_4_}Mg_2_ MOF (a) to give {Cu_6_Ni_4_}Ni_2_ (b), the exchange of the Ni^2+^ cations of the pores by [Pd^II^(NH_3_)_4_]_2_ yielding {Cu_6_Mg_4_}Pd_2_ (c) and the final reduction process affording [Pd_4_]_0.5_@Na_3_{Ni^II^_4_[Cu^II^_2_(Me_3_mpba)_2_]_3_} (d). (e) and (f) show the perspective views of a channel of the MOF containing the [Pd_4_]^2+^ clusters and a proposed formation mechanism for them. Reproduced from ref. 179 with permission.

With a drive to smaller sub-nanoscale MNC being targeted for their potential catalytic properties, characterisation becomes a greater challenge and strategies that allow MNC synthesis *via* SC–SC processes become important. In connection with this challenge, Pardo *et al.* reported the gram-scale preparation of tetranuclear naked Pd_4_ clusters, with mixed-valence 0/+1 oxidation states, within the channels of an anionic MOF yielding a composite with formula [Pd_4_]_0.5_@Na_3_{Ni^II^_4_[Cu^II^_2_(Me_3_mpba)_2_]_3_} [where Me_3_mpba = *N*,*N*′-2,4,6-trimethyl-1,3-phenylenebis(oxamate)].^179^ The robustness and high crystallinity of the selected anionic MOF allows it to maintain its crystallinity even after three consecutive PSM processes that include a transmetallation of the framework, the insertion of the Pd^2+^ cations within the channels of the MOF and the final reduction with NaBH_4_ to form the ligand-free [Pd_4_]^2+^ cations, supported on the walls of the MOF (Fig. 9). The anionic MOF plays a key role in the stabilisation, characterisation and formation of these low-valent MNCs. Pd^2+^ cations are introduced within the MOF channels, replacing Ni^2+^, *via* a cation exchange process. As a consequence, there are a limited number of Pd^2+^ cations, which are homogeneously distributed along the channels (Fig. 9(c)). These two points undoubtedly contribute to the controlled formation of such small well-defined tetranuclear Pd NCs, which are further stabilised by the framework (Fig. 9(e)) and remarkably visualised by SCXRD. The resulting heterogeneous solid catalyst outperformed the “state-of-the-art” catalysts in different carbene-mediated reactions of diazoacetates, showing high yields (>90%) and turnover numbers close to 10^6^, with good recyclability.

**Fig. 10:**
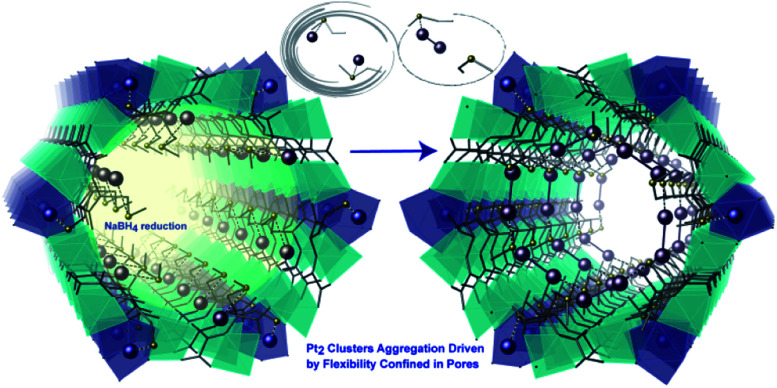
Detailed comparison of crystal structures of a single pore of the MOFs after the Pt^2+^ inclusion (left) and after the reduction process (right) underlining the proposed mechanism to generate the Pt_2_ clusters.Reproduced from ref. 182 with permission.

The previous example shows how the use of an anionic MOF can be extremely helpful to control the number of metal atoms that can be inserted within the channels and to retain them at specific positions within this confined space. These two points, together with the presence of stabilising interactions between the network and the species formed, are at the origin of the successful, controlled, formation and direct structural characterisation of the Pd_4_ NCs. The same authors designed another highly crystalline and functional amino acid derived MOF, whose pores were densely decorated with thio-alkyl groups, capable of interacting and retaining a variety of metals^180,181^ and clusters, using the well-known affinity of sulfur for soft metals. Thus, by following a similar solvent assisted impregnation method, consisting of the suspension of the MOF in solutions containing Pt^2+^ cations, followed by subsequent reduction using NaBH_4_, well defined Pt^0^_2_ dimers can be embedded within the MOF channels, retained by the thio–ether appendages.^182^ The resulting hybrid material, with formula (Pt^0^_2_)_0.5_(Pt^II^Cl_2_)@{Ca^II^Cu^II^_6_[(*S*,*S*)–methox]_3_(OH)_2_(H_2_O)} [methox = bis[(*S*)-methionine]oxalyl diamide], showed a high loading of platinum atoms (*ca*. 17 wt%). The nature of the Pt^0^_2_ NCs could be determined by combining HAADF-STEM and, again, SCXRD (Fig. 10) allowing resolution of the crystal structure of the MNCs. The presence of the sulphur-containing groups, capable of anchoring the Pt^2+^ cations and restricting their number within the pores, is important, and allows snapshots of the formation process to be garnered with the help of SCXRD. The reactivity of this MNC decorated MOF (Pt^0^_2_)_0.5_(Pt^II^Cl_2_)@{Ca^II^Cu^II^_6_[(*S*,*S*)–methox]_3_(OH)_2_(H_2_O)}, was shown by examining low temperature catalysis (25 to 140 °C) of energetically–costly industrial reactions in the gas phase, such as hydrogen cyanide (HCN) production, carbon dioxide (CO_2_) methanation and alkene hydrogenations.^182^

**Fig. 11:**
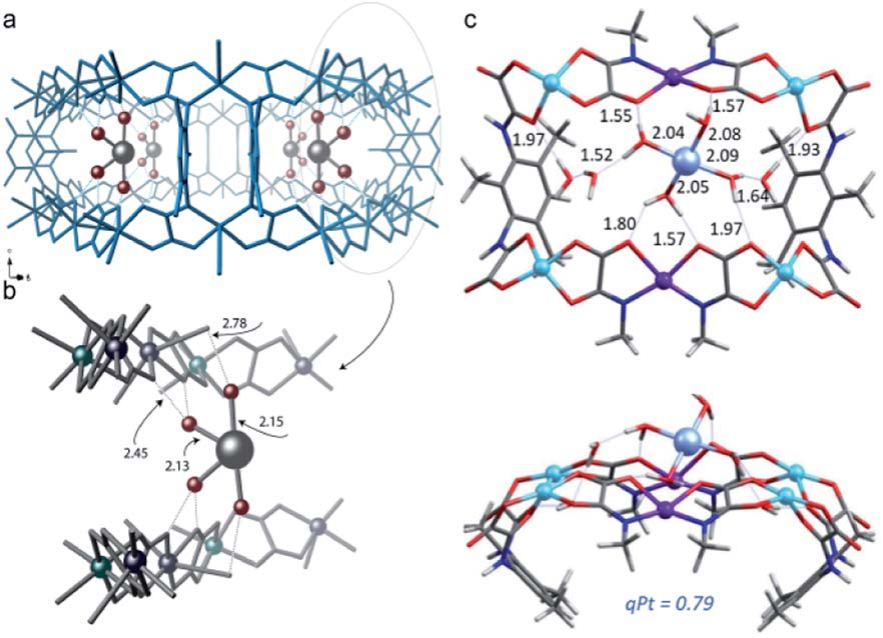
(a and b) Detailed SC-XRD results of the Pt_1_^1+^ complex within the MOF. (c) DFT optimized structure of the Pt_1_^1+^ complex with seven water molecules. Reproduced from ref. 183 with permission.

The same strategy can be extended for the MOF-driven preparation of highly reactive, coordinatively unsaturated single atoms. Pardo *et al.* reported the gram-scale preparation of platinum single atoms^183^ following the same strategy and within the same MOF used for the previously described [Pd_4_]^2+^ mixed-valence NCs. Indeed, it was recognised that the anionic MOF {Ni^II^_4_[Cu^II^_2_(Me_3_mpba)_2_]_3_} seems to have unique characteristics for the stabilisation of low-valent species such as the aforementioned mixed-valence 0/+1 Pd_4_ MNCs^179^ and single Pt atoms. Thus, SCXRD studies demonstrated that Pt_1_^1+^ single atoms could be stabilized in a confined space by a well-defined first water sphere and by a second coordination sphere linked to the MOF through electrostatic and H-bonding interactions (Fig. 11). These observations were further confirmed by theoretical calculations. The resulting low valent Pt_1_^1+^ single-atom, which is difficult to obtain outside the MOF, showed superior catalytic performance for the water water–gas shift reaction (WGSR: CO + H_2_O → CO_2_ + H_2_) at lower temperatures than conventional strategies.

In contrast to the detailed structural analysis reported in the partially flexible amino-acid derived MOFs of Pardo, achieving such accurate structural insight is more difficult in rigid materials. A concomitant example concerning the confinement of Pt single atoms within MOFs was reported, by the group of Jiang.^184^ In this example, the selected MOF was the robust aluminium-based MOF Al-TCPP, (AlOH)_2_H_2_TCPP [where H_2_TCPP = 4,4′,4′′,4′′′-(porphyrin-5,10,15,20-tetrayl)tetrabenzoate)]. The synthetic strategy involved immersion of the MOF in a solution of K_2_PtCl_4_ followed by reduction to Pt^0^. The Pt atoms, which could not be characterised by SCXRD due to low crystallinity of the original MOF, are confined within the porphyrin rings. Consequently, there is a homogenous distribution of Pt atoms throughout the MOF, with their specific positions determined by the porphyrin rings, as inferred from a number of spectroscopic and diffraction analyses. These two examples further highlight the importance of moderate structural flexibility in allowing SCXRD of matrix isolated species.

## 5. Conclusions and outlook

Metal–Organic Frameworks are a highly versatile platform for the isolation and characterisation of reactive species. In this perspective we have highlighted how the MOF pore space, metal nodes and organic linkers can each be used to stabilise unusual molecular coordination environments or nanocluster architectures and thus allow for the exploration of their chemistry. However, significant scope for the development of MOFs as a stabilising matrix remains. As many of the examples we have canvassed show, MOF crystallinity is a salient feature that allows for precise structural determination of species that can be difficult to elucidate *via* spectroscopic methods.

In most cases the motivation to generate reactive moieties within MOFs is to enhance a specific performance characteristic such as gas adsorption or catalysis. However, exploiting these systems to advance our fundamental understanding of metal-based reaction mechanisms offers considerable promise, especially with respect to heterogeneous conditions. For example, within the MOF pore environment it is possible to carry-out synthetic procedures in the absence of solvent or under carefully controlled conditions. Thus, it is conceivable that a highly reactive coordinatively and electronically unsaturated metal centre could be generated and structurally characterised. Access to such moieties would allow researchers to probe the first steps of challenging reactions.

However, significant challenges remain and the use of MOFs to isolate reactive species is not ubiquitous. For the effective use of this strategy retention of MOF crystallinity is mandatory. Research in this area suggests that to support maintenance of crystallinity after consecutive chemical reactions in the MOF pores, structural flexibility is required to facilitate changes in bond lengths and coordination geometries at the metal site. Whilst MOF flexibility is advantageous it is also clear that the MOF must be sufficiently stable to survive the reaction conditions associated with the reactions under investigation. This combination of properties requires careful appreciation of the MOF design and can set challenging constraints upon the systems that can be effectively used. When one considers the further requirement for low symmetry and a density of guest metal sites that does not engender steric crowding then it is clear that there are limitations upon the variety of MOFs that can be employed for such studies.

Given the limitations identified throughout the perspective, we have tried to articulate a set of “best practices” to enable others to utilise the molecular architectures of MOFs to isolate and structurally characterise reactive metal-based species. However, we acknowledge that there is an element of trial and error and, from our own undertakings, are aware that significant work goes into perfecting the isolation conditions that allow retention of crystallinity. In terms of accessing novel metal-based species, the node-based approach (Section 4a) offers significant advantages due to the vast array of metal nodes and clear evidence that many of these can be chemically manipulated by metal substitution and subsequent ligand coordination. However, MOF nodes typically occupy high symmetry crystallographic positions, and furthermore, post-synthesis substitution usually yields low levels of metal substitution (often distributed across multiple sites); thus SCXRD characterisation of these species is highly challenging. Accordingly, the application of this technique to lower symmetry MOFs is likely to be more fruitful in providing structure determination of reactive metal entities. Additionally, strategies to achieve quantitative metalation of the target site, without loss of crystallinity, are vital. In cases where diminished crystallinity is a significant problem, we have found that slowing down the post-metalation step can be beneficial. This can be achieved by using exchange solvents where the added metal is sparingly soluble, or by conducting the chemistry at lower temperatures.

Matrix isolation and structure determination using the pre-metalation approach has been more successful than other approaches once the challenge of crystallising a MOF with the selected pre-metalated linker has been overcome. Most of the examples reviewed were relatively low symmetry MOFs which aids structural characterisation. However, the main difficulties with this approach are (i) the high density of metalated sites reduces the cavity and channel dimensions, which limits access to the metal species, and (ii) MOFs that are synthetically compatible with pre-metalated linkers are commonly composed of metal nodes of, relatively, low-stability.^136^

For the post-synthetic metalation approach, there are several systems that allow SCXRD to be used for characterisation of the initially post-metalated MOF, but relatively few that allow subsequent reactions of the tethered metal to be characterised. The MnMOF-1 platform, used by some of the authors and discussed above, does enable both post-metalation and subsequent SCXRD characterisation of reaction products. We contend that the success of MnMOF-1 is due to a number of factors, including the ease of quantitative metalation (low tethered metal site density; the plate-like morphology of the crystals), the low symmetry structure, and a degree of flexibility at the metal-binding site. However, this platform is still prone to losses in crystallinity and so careful attention must be given to the solvents used for metalation^140^ and the rate of the post-synthetic metalation (see previous points about introducing the extraneous metal to the metal nodes). One issue with the post-synthetic metalation approach is that the types of anchoring sites, and in particular the types of ligand donors are fairly limited; however, new materials with phosphines and arsine donors, for example, are now being investigated.^138,139^ A further difference between the pre-metalated and post-metalated systems is that the latter require open pores needed for post-synthetic metalation; this can be both an advantage, by allowing reactants to access the tethered metal site, but also a disadvantage as the pores provide pathways for co-ligand loss and decomposition.

Finally, using host–guest chemistry to isolate and structurally characterise organic guests within the MOF pores, including those that are liquids or transiently stable, has become relatively well developed and this approach is starting to be applied to metal-based species. For example, work by one of the co-authors using amino acid derived MOFs provides access to a more hydrophilic set of crystalline sponges. Nevertheless, trial and error approaches to identifying the best MOF host are still employed. In this sense, apart from the need to enhance both the creation of novel MOF databases^185^ and the use of theoretical calculations^186^ to predict the most appropriate MOF to host each guest species, it seems mandatory to further explore the development of new MOF-generation algorithms^187^ to facilitate the selection – *via* computational screening – of the most appropriate MOFs.

The scope for using MOFs as an ordered molecular scaffold to isolate and structurally elucidate metal-based species is vast. It can be anticipated that the application of this technique will realise unprecedented insight into reaction processes and the structural elucidation of highly unusual, and reactive species. Clearly, overcoming the hurdles associated with MOF design are worth the rich rewards that this chemistry affords.

## Conflicts of interest

There are no conflicts to declare.
